# Epstein-Barr Virus in Gastric Carcinoma

**DOI:** 10.3390/cancers6042259

**Published:** 2014-11-07

**Authors:** Jun Nishikawa, Hironori Yoshiyama, Hisashi Iizasa, Yuichi Kanehiro, Munetaka Nakamura, Junichi Nishimura, Mari Saito, Takeshi Okamoto, Kouhei Sakai, Yutaka Suehiro, Takahiro Yamasaki, Atsunori Oga, Hideo Yanai, Isao Sakaida

**Affiliations:** 1 Department of Gastroenterology and Hepatology, Yamaguchi University Graduate School of Medicine, Minami-Kogushi 1-1-1, Ube, Yamaguchi 755-8505, Japan; E-Mails: munetaka@yamaguchi-u.ac.jp (M.N.); nishimuj@yamaguchi-u.ac.jp (J.N.); mari-s@zj8.so-net.ne.jp (M.S.); tokamoto@yamaguchi-u.ac.jp (T.O.); sakaida@yamaguchi-u.ac.jp (I.S.); 2 Department of Microbiology, Shimane University Faculty of Medicine, 89-1 Enyacho, Izumo City, Shimane 693-8501, Japan; E-Mails: yosiyama@med.shimane-u.ac.jp (H.Y.); iizasah@med.shimane-u.ac.jp (H.I.); kanehiro@med.shimane-u.ac.jp (Y.K.); 3 Department of Oncology and Laboratory Medicine, Yamaguchi University Graduate School of Medicine, Minami-Kogushi 1-1-1, Ube, Yamaguchi 755-8505, Japan; E-Mails: sakaik@yamaguchi-u.ac.jp (K.S.); ysuehiro@yamaguchi-u.ac.jp (Y.S.); t.yama@yamaguchi-u.ac.jp (T.Y.); 4 Department of Pathology, Yamaguchi University Graduate School of Medicine, Minami-Kogushi 1-1-1, Ube, Yamaguchi 755-8505, Japan; E-Mail: oga@yamaguchi-u.ac.jp; 5 Department of Clinical Research, National Hospital Organization Kanmon Medical Center, 1-1 Sotoura, Chofu, Shimonoseki, Yamaguchi 752-8510, Japan; E-Mail: yanaih@kanmon-mc2.hosp.go.jp

**Keywords:** Epstein-Barr virus, gastric carcinoma, DNA methylation

## Abstract

The Epstein-Barr virus (EBV) is detected in about 10% of gastric carcinoma cases throughout the world. In EBV-associated gastric carcinoma, all tumor cells harbor the clonal EBV genome. Gastric carcinoma associated with EBV has distinct clinicopathological features, occurs predominately in men and in younger-aged individuals, and presents a generally diffuse histological type. Most cases of EBV-associated gastric carcinoma exhibit a histology rich in lymphocyte infiltration. The immunological reactiveness in the host may represent a relatively preferable prognosis in EBV-positive cases. This fact highlights the important role of EBV in the development of EBV-associated gastric carcinoma. We have clearly proved direct infection of human gastric epithelialcells by EBV. The infection was achieved by using a recombinant EBV. Promotion of growth by EBV infection was observed in the cells. Considerable data suggest that EBV may directly contribute to the development of EBV-associated GC. This tumor-promoting effect seems to involve multiple mechanisms, because EBV affects several host proteins and pathways that normally promote apoptosis and regulate cell proliferation.

## 1. Introduction

The Epstein-Barr virus (EBV) is associated with a variety of tumors derived from B cells, T cells, natural killer (NK) cells, and epithelial cells. Burkitt lymphoma [1], post-transplant lymphoproliferative disease [2], and Hodgkin’s disease [3] are B-cell tumors. Peripheral T-cell lymphomas [3] and NK/T-cell lymphomas are T-cell tumors and NK-cell tumors, respectively. Nasopharyngeal carcinoma [1] and gastric carcinoma (GC) [3] are epithelial tumors.

Existence of the EBV genome in GCs was first detected in 1990 by Burke *et al.* using the polymerase chain reaction (PCR) technique [4]. Since then, about 10% of GCs have been identified as EBV positive. In each EBV-positive case of GC, almost all carcinoma cells are infected with the virus [5,6], and tumor cells exist as a monoclonal proliferation of EBV-infected cells [7,8]. These facts suggest the significance of EBV in the development of GCs.

Gastric cancer is the second leading cause of cancer-related deaths globally, and 60% of these deaths occur in East Asia, which includes Japan [9]. The worldwide occurrence of EBV-associated GC is estimated at more than 50,000 cases per year [10]; therefore, EBV-associated GC is the most common cancer among EBV-related malignancies.

## 2. Definition

**Figure 1:**
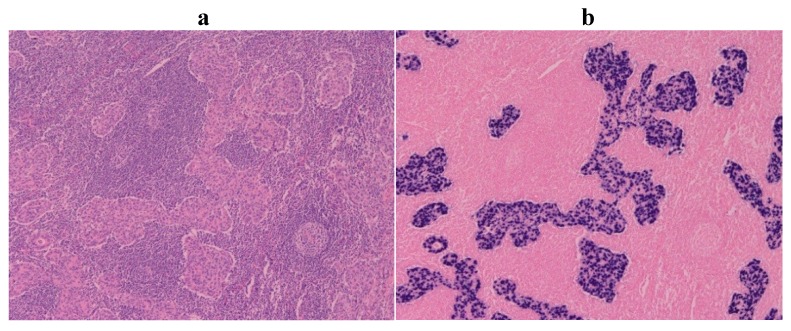
Lymphoepithelioma-like subtype of Epstein-Barr virus (EBV)-associated gastric carcinoma. (**a**) H & E staining; (**b**) EBV-encoded small ribonucleic acid 1 (EBER1) *in situ* hybridization demonstrates positive nuclei in the carcinoma cells, which are surrounded by infiltrating lymphocytes.

In addition to the detection of the EBV genome in GCs using PCR [4], EBV-encoded small RNA 1 (EBER1) was also detected using *in situ* hybridization (ISH). Various studies in the early 1990s indicated that EBV-associated GC comprises about 10% of all GCs worldwide [5,6,7,8]. EBER1 is highly abundant (10 million copies per cell) in individual infected cells. Typically, EBER1 can be detected in the nuclei of tumor cells; however, the EBER1 signal is negative in reactive lymphoid infiltrate cells or normal gastric mucosa cells (Figure 1). To make the diagnosis of EBV-associated GC before treatment, EBER1-ISH should be applied to gastric mucosal biopsy samples from patients who have undergone upper gastrointestinal endoscopy. Patients with EBV-associated GC have elevated levels of serum antibodies against EBV early antigen and EBV capsid antigen. However, EBV nuclear antigen (EBNA) 1 antibody titers do not show significant difference between patients and healthy counterparts [7].

## 3. Epidemiology

GC is one of the most common malignancies in Japan. Among the various histological types from Japanese gastric cancer cases, the incidence of EBV-positive cases was 7.0% in 1994 [7]. In contrast to Burkitt lymphoma and nasopharyngeal carcinoma, which are distributed endemically in equatorial Africa and Southeast Asia, respectively, EBV-associated GC is distributed worldwide in a similar proportion [10]. Regional difference in the incidence of EBV-associated GC is also reported. The incidence of EBV-associated GC in all cases of gastric cancer ranges from a high of 16%–18% in the USA and Germany to a low of 4.3% in China. The regional difference in the incidence of EBV-positive cases in gastric cancers indicates that the prevalence EBV-associated GC is inversely related to the incidence of GC [11].

EBV-associated GC has distinct clinicopathological features, is present predominately in men and in younger-aged individuals, and presents a generally diffuse histological type [12,13]. Most studies have not shown evident age dependence in the frequency of EBV-associated GC. Almost all of the studies showed male predominance of EBV-associated GC, suggesting that risks related to lifestyle or occupational factors may exist among males [14]. An interview study in Japan showed that salty food intake and exposure to wood dust and/or iron filings, which may induce mechanical injury to the gastric epithelia, are related to a higher risk of EBV-associated GC [15]. Camargo *et al.* recently showed that the association of smoking with gastric cancer is stronger for EBV-positive than EBV-negative tumors [16].

## 4. Pathology

EBV-associated GC has definite histological relevance to GC with lymphoid stroma (GCLS) [17,18,19], which was originally described by Watanabe *et al.* as a subtype of the carcinoma [20]. GCLS is a poorly differentiated adenocarcinoma with diffuse and intense lymphocyte infiltration similar to EBV-associated nasopharyngeal lymphoepithelioma. More than 80% of lymphoepithelioma-like GC is infected with EBV [17,18,19] (Figure 1), whereas ordinary-type GC, comprising 5%–10% of all cases of GC, shows features of moderately or poorly differentiated adenocarcinoma with various degrees of lymphocytic infiltration. Further infiltration of the carcinoma (tumor cells) into the submucosa is occasionally accompanied by EBV-associated GC generally exhibiting a characteristic histology referred to as GCLS [21].

EBV-associated GC has a null or gastric phenotype as determined by the expression pattern of the mucin molecules MUC5AC and MUC6 [22,23] and is characterized by a relative lack of intestinal phenotypic expression, including Cdx2 [24]. According to these findings, the targets of EBV infection and their subsequent transformation are seemingly the precursor cells possessing intrinsic differentiation potential toward the gastric cell type.

## 5. Clinical Features

**Figure 2:**
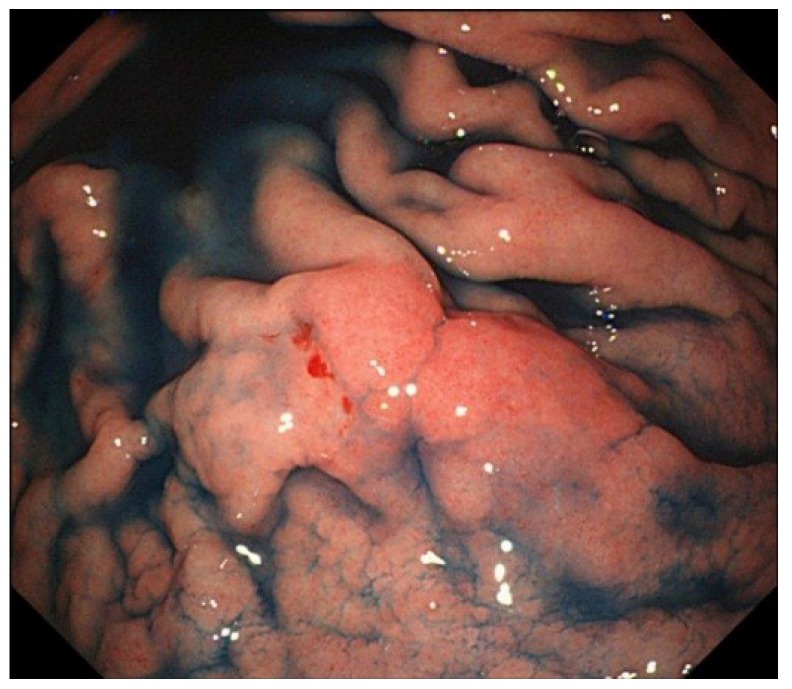
Endoscopic image of an Epstein-Barr virus-associated gastric carcinoma in the upper gastric body. The tumor shows a protruded shape probably because of the abundant lymphocyte infiltration.

The most useful modality for the diagnosis of GC is endoscopy. In one analysis, 124 GCs from 117 patients were examined by EBER1-ISH. Of the 124 tumors, 12 (9.7%) were identified as EBV-associated tumors [25]. It is of note that EBV-associated GC predominantly localizes in the non-antrum part of the stomach (Figure 2) and appears as superficial depressed or ulcerated lesions. A histological feature of EBV-associated GC is a diffuse-type carcinoma accompanied by abundant lymphocyte infiltration (*i.e.*, GCLS). In some patients, endoscopic ultrasound reveals a hypoechoic mass in the third hyperechoic layer reflecting submucosal nodules of lymphoid stroma, which is composed of carcinoma cells and infiltrating lymphocytes [26].

It is known that *Helicobacter pylori* is strongly related to cancer and is an etiological agent of chronic gastritis and intestinal metaplasia. It is distinctive that *H. pylori*-related gastritis frequently initiates in the antrum. In the case of EBV-associated GC, tumors are frequently located near the mucosal atrophic border, where mild to moderate atrophy is common [27]. We have frequently detected both EBV and *H. pylori* in the mucosa of patients with moderate chronic atrophic gastritis, where inflammatory cell infiltration is abundant, and not in the mucosa with marked atrophic gastritis, where inflammatory cell infiltration is scarce [28].

Gastric remnant cancer arises after distal gastrectomy for benign disease, which includes refractory gastric or duodenal ulcer disease and recurrent ulcer with gastric outlet obstruction. The incidence of gastric remnant cancer ranges from 1% to 7% of all GCs and is still increasing [29]. Gastric remnant carcinoma is frequently (25% to 41.2%) associated with EBV infection. It is considered that the reflux of bile and pancreatic juice causes regenerative atypia and cell proliferation in epithelial cells [30]. Atrophic change of remnant gastritis in Billroth-II anastomoses is frequently accompanied by EBV-positive gastric remnant carcinoma [31]. Gastritis cystica polyposa, frequently observed in the remnant stomach, is a suspected precursor lesion of EBV-associated GC, but no direct evidence of EBV infection in these lesions has been found [32].

## 6. Treatment and Prognosis of EBV-Associated GC

The current therapy for EBV-associated GC does not use any special methods. Because undifferentiated-type cancer is prevalent in EBV-associated GC, most of these tumors are removed by surgical resection. Early EBV-associated GC has a low frequency of lymph node metastasis. Endoscopic treatment can be applied in such cases. The authors experienced a case of early EBV-associated GC with submucosal invasion in which palliative endoscopic treatment was performed. No recurrence was observed in the patient for more than 4 years [33]. A clinicopathological study in The Netherlands showed that EBV-associated GC has a significantly low frequency of lymph node metastasis compared with EBV-negative stomach cancer, resulting in a better prognosis than that with the EBV-negative cases [34]. A recent meta-analysis revealed that EBV-associated GC showed an infrequent tendency toward lymph node metastasis. After adjustment for TNM stage and other prognostic indicators, EBV positivity was associated with lower mortality [35]. Further studies are needed to identify the mechanisms underlying this prognostic association.

## 7. Growth-Promoting Effects of EBV in Epithelial Cells

### 7.1. Models of EBV Infection of Gastric Epithelial Cells

EBV infects both B lymphocytes and epithelial cells because the virus has been discovered in Burkitt lymphoma cells, Hodgkin cells, nasopharyngeal carcinoma cells, and GC cells. Experimental EBV infection of B cells is very efficient because EBV uses CD21, a high-affinity receptor, for its entry into the cell [36,37]. However, epithelial cells are CD21 negative, and infection of epithelial cells could not be achieved for a long time, not until CD21 expression was overcome by gene transfer [38,39]. Infection of EBV with human gastric epithelial cells was experimentally proved by our group [40], and EBV-infected gastric cells (AGS) have been established by Marquitz *et al.* [41]. A recombinant EBV with a neomycin resistance gene [42,43] was used for epithelial infection, and thus, epithelial cells, which do not express a CD21 EBV receptor, could be infected with EBV. This infection of CD21-negative epithelial cells was not blocked by anti-CD21 monoclonal antibody [40]. Next, EBV was efficiently transferred to epithelial cells by mixing epithelial cells with recombinant EBV-producing B cells [44]. There are several epithelial cell lines, such as CNE1 and HONE1, which can achieve experimental infection with EBV. Instead of these cell lines, SNU-719 cells [45], NCC24 cells [46], and KT cells [47] are a few of the cell types that retain the same clonal EBV genome and the pattern of EBV gene expression (type I EBV) as that in the original tumor biopsy. The KT cell is a good *in vivo* model of EBV-associated GC and expresses high IL-1β compared with EBV-negative gastric tumor cells [48].

### 7.2. Growth-Promoting Effects of EBV

EBV immortalizes B cells *in vitro*. EBNA 2 and latent membrane protein 1 (LMP1) appear to play the most important roles in the immortalization of lymphocytes. However, they are not expressed in EBV-associated GC, raising doubts about the importance of the presence of EBV. We attempted to infect gastric primary culture cells with EBV [49]. Primary gastric epithelial cells from healthy gastric mucosal biopsies were infected with recombinant EBV carrying a neomycin resistance gene, and infected cells were selected for using G418. As a result, we repeatedly separated cell clones that could be maintained for at least 300 generations. The selected EBV-infected cells expressed Qp-driven EBNA 1, EBER, BARTs, and latent membrane protein 2A (LMP2A). The pattern of latent gene expression was similar to EBV-associated GC. The EBV-infected clones had higher proliferation rates and at least twice the cell saturation density compared with non-infected clones into which the neomycin resistance gene had been introduced as a control, and the malignant phenotype was confirmed by colony formation in soft agar and tumorigenicity in SCID mice. EBV infection also promoted growth of gastric cancer cell lines NU-GC-3 and AGS [41,49].

## 8. Virus and Host Interactions at the Molecular Level

### 8.1. Genetic Alterations in EBV-Associated GC

In EBV-associated GC, studies of genetic alteration are limited. Van Rees *et al.* [50] and Chong *et al.* [51] reported that chromosomal losses were extremely rare in EBV-associated GC in contrast to the high frequency in EBV-negative GC. Chromosomal aberrations in EBV-associated GCs were globally tested by comparative genomic hybridization. Zur Hausen *et al.* showed that loss of chromosomes 4p, 11p, and 18q was distinct in EBV-associated GCs [52]. 18q harbors the DCC and SMAD4 genes, which are known tumor-suppressor genes. Chan *et al.* reported that gains in chromosome 11 copy numbers are common in EBV-associated malignancy including EBV-positive GC, lung cancer, and lymphoma [53]. As well, microsatellite instability is not common in EBV-associated GC [54]. Similarly, p53 mutation and overexpression are not frequent in EBV-associated GCs [55,56]. These findings indicate that genetic abnormality is not the major pathway to the development of EBV-associated GC.

### 8.2. DNA Hypermethylation in EBV and Host Genomes

Methylation of the tumor suppressor gene is a key abnormality in EBV-associated GC [57,58,59]. In tumor cells of EBV-associated GC, CpG island methylation is frequently observed at promoters of various tumor-related genes, which must take important parts in the development and progression of gastric cancer [60]. Methylation frequencies of several tumor suppressor genes, APC, PTEN, and RASSF1A, and cell adhesion molecules, THBS1 and E-cadherin, were reported to be significantly higher in EBV-associated GC [61,62,63]. Because demethylation agents induce lytic EBV infection in latently EBV-infected cells followed by apoptotic cell death, the therapeutic application of demethylating agents may lead to the lysis of cancer cells [64]. These facts strongly support possible application of demethylating agents to the medical treatment of EBV-associated GC.

We compared methylation status between EBV-associated GCs and EBV-negative controls whose age, sex, histology, depth of invasion, and stage were matched. EBV-associated GCs showed higher methylation frequencies in 12 of 16 tumor-related genes compared with EBV-negative controls. The frequency of methylation at 6 specific loci (MINT2, MINT31, p14, p16, p73, and RUNX3) was significantly higher in EBV-associated GCs than in EBV-negative controls [65]. Moreover, the DNA methylation status in the naturally derived EBV-positive gastric adenocarcinoma cell line SNU-719 was also examined by the method of methylated CpG island recovery on chip assay [66]. Expression of several genes was regulated by DNA methylation in EBV-associated GC. The methylation frequencies of p73, BLU, FSD1, BCL7A, MARK1, SCRN1, and NKX3.1 were significantly higher in EBV-associated GC than in EBV-negative GC [66].

The precise molecular mechanism that induces host DNA methylation during the early stage of EBV infection of the gastric epithelium is not fully understood. LMP2A is reported to induce the phosphorylation of STAT3, which activates DNA methyltransferase 1 (DNMT1) transcription and causes loss of PTEN expression through CpG island methylation of the PTEN promoter in EBV-associated GC [67]. However, LMP2A is not expressed in every case of EBV-associated GC [68], and EBV-associated GC patients are usually negative for LMP2A antibody [69]. LMP1 can also induce aberrant DNA methylation by activating DNMT1 through the JNK signaling pathway [70] and inducing DNA methylation of host cells [71]. However, LMP1 is scarcely expressed, and LMP1 protein is generally absent in EBV-associated GC [72]. Methylation of similar genes has been reported in hepatitis B and C [73,74], suggesting that there must be a common mechanism underlying the formation of infection-associated cancers.

**Figure 3:**
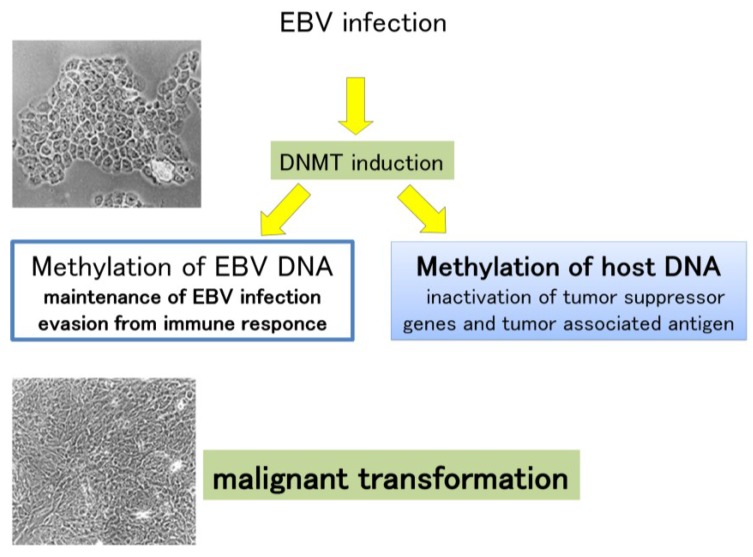
Aberrant DNA methylation might lead to the development and progression of Epstein-Barr virus (EBV)-associated gastric carcinoma. DNMT, DNA methyltransferase.

The status of DNA methylation in the EBV genome was intensively investigated [75]. The expression of EBV latent genes is strictly regulated through viral DNA methylation in EBV-infected cells. The Cp/Wp EBNA promoters are known to transcribe all EBNAs. However, in Burkitt lymphoma and nasopharyngeal carcinoma, the Cp/Wp promoters are methylated and the only EBNA1 promoter, Qp, is used instead [76,77]. Moreover, in EBV-positive nasopharyngeal carcinoma, LMP1 expression is down-regulated by methylation in its promoter region [78]. The pattern of latent gene expression in EBV-positive GCs is similar to that of Burkitt lymphoma, in which only Qp is actively used [7]. These results indicate that the methylation status of the EBV genome regulates the pattern of latent gene expression in EBV-positive tumor cells. Because methylation occurs on viral DNA in EBV-associated GC cells, methylation of host cell DNA may also occur, for example, on tumor suppressor genes that regulate the cell cycle and apoptosis. Aberrant DNA methylation might occur in EBV-positive cells, thus promoting the development and progression of EBV-associated GC (Figure 3).

### 8.3. EBV Latent Genes and Host Interaction

Iwakiri *et al.* reported that EBV infection promoted growth of gastric cancer cells by increased production of insulin-like growth factor (IGF)-1 as an autocrine growth factor. It was also revealed that EBERs are responsible for the induction of IGF-1 [79]. The oncogenic role of EBERs has been reported for inhibition of apoptosis in the human epithelial cell line Intestine 407 [80]. EBER was found to bind double-stranded RNA-dependent protein kinase R, an interferon-inducible serine/threonine kinase, and abrogate its kinase activity. These results indicate that EBER contributes to the maintenance of epitheloid malignancy.

The oncogenic role of other genes such as BARF1 (BamHI A rightward open reading frame 1) [81] and LMP2A [67,82] has also been reported. The expression of the EBV-encoded oncogene BARF1 has been reported in EBV-associated GC. Wiech *et al.* reported that cyclin D1 is induced in BARF1-transfected epithelial cells and is overexpressed in EBV-associated GC [83]. LMP2A is reported to inhibit transforming growth factor-b1-induced apoptosis in a GC cell line [84]. Recently, it was demonstrated that LMP2A upregulated cellular survivin gene expression through the nuclear factor-kB pathway in GC cell lines with EBV infection [82]. In addition, LMP2A upregulates cellular DNMT1 in EBV-associated GC through the phosphorylation of STAT3, causing promoter hypermethylation of a tumor suppressor gene, PTEN [67].

### 8.4. EBV microRNA and Gastric Cancer

A microRNA (miRNA) is a small (20 to 25 nucleotides) non-coding RNA derived from double-stranded RNAs, which functions in RNA silencing and post-transcriptional regulation of gene expression. miRNA is incorporated into the RNA-induced silencing complex (RISC) in cytosol, binds to the 3' un-translated region (UTR) of mRNA, and then silences translation by destabilizing mRNA through shortening of its poly A tail [85]. miRNA is found in plants, animals, and some viruses. EBV is one of the first viruses reported to contain viral miRNA, the genome of which codes 25 miRNA precursors and produces 44 kinds of different miRNAs [86,87]. A number of mRNA targets by EBV miRNAs have been reported mainly in B lymphocytes via the bioinformatics approach [88,89]. Recent results of EBV miRNA targets using gastric epithelial cells are introduced in this section.

Choy *et al.* reported on the regulation of p53 up-regulated modulator of apoptosis (PUMA) by an EBV miRNA, miR-BART5-5p, which is abundantly expressed in nasopharyngeal carcinoma and EBV-associated GC cells [90]. Marquitz *et al.* showed that in vitro infection of an AGS cell line with EBV alters the growth properties of the cells and induces growth in soft agar in accordance with high levels of expression of the BamHI A rightward transcript (BART) miRNAs [41]. They showed downregulation of a tumor suppressor gene, PTEN, cellular adhesion proteins, integrin alpha 5 and alpha V, and signal transducer STAT6. These results suggested that the expression of EBV miRNA highly influences the genesis of EBV-associated GC. Choi *et al.* also investigated an AGS cell line and reported that the 3' untranslated region of baculovirus inhibitor of apoptosis repeat-containing ubiquitin-conjugating enzyme (BRUCE) was affected by EBV miR-BART15-3p [91]. miR-BART miRNAs target many other anti-apoptotic genes; however, the precise roles of each gene for tumor formation are still not well understood.

Many research groups reported expression of EBV miRNAs in gastric cancer cells and histological samples from gastric cancers [92,93]. It is of note that YCCEL1 and SNU-719 cell lines are derived from a gastric cancer patient, respectively, and maintain viral episomes. Seemingly, this is the reason why these cell lines show expression profiles of EBV miRNAs similar to samples from gastric cancer patients [94,95,96]. These two cell lines are expected to become important tools for the study of EBV miRNA.

## 9. Summary

Considerable data suggest that EBV can increase cell proliferation and survival; and through these effects; EBV may directly contribute to the development of EBV-associated GC. This tumor-promoting effect seems to involve multiple mechanisms; because EBV affects several host proteins and pathways that normally promote apoptosis and regulate cell proliferation.
